# Inhibitors of metalloprotease, γ-sectretase, protein kinase C and Rho kinase inhibit wild-type adenoviral replication

**DOI:** 10.1371/journal.pone.0236175

**Published:** 2020-07-22

**Authors:** Alice Liu, Cristhian J. Ildefonso, Wesley S. Bond, Mary Y. Hurwitz, Richard L. Hurwitz

**Affiliations:** 1 Department of Pediatrics, Baylor College of Medicine, Houston, Texas, United States of America; 2 Summer Undergraduate Research Training Program, Baylor College of Medicine, Houston, Texas, United States of America; 3 Translational Biology and Molecular Medicine Program, Baylor College of Medicine, Houston, Texas, United States of America; Sechenov First Medical University, RUSSIAN FEDERATION

## Abstract

Adenoviruses cause upper respiratory infections, conjunctivitis, keratitis, and gastrointestinal illness. These can be fatal in immunocompromised individuals. Adenoviruses have also been engineered into viral vectors to deliver therapeutic genes or induce immunity as vaccine carriers. The success of ocular gene therapy is driven partly by the immunologic and biochemical influences of the intraocular environment. We have shown that versican and hyaluronan modulate adenoviral vector transgene expression through CD44 signaling. Herein we explored the role of these pathways on virus replication and viral protein expression of wild type adenovirus. We report that the addition of vitreous humor (which contains both versican and hyaluronan) increases viral hexon protein levels. Vitreous humor also increased wild type adenovirus DNA replication *in vitro*. Metalloproteinase and γ-secretase inhibitors, which inhibit CD44 proteolytic activation, blocked adenoviral replication *in vitro*. Similarly, protein kinase C and RhoA kinase inhibitors, both proteins associated with CD44 mediated pathways, also inhibited wild type adenoviral replication *in vitro*. Application of metalloproteinase and γ-secretase inhibitors to human conjunctival explants sharply decreased adenoviral vector gene expression. Our results demonstrate that pharmacologic delivery of these inhibitors is easily achievable. The inhibition of these enzymes should be explored as potential therapies of wild type adenoviral infections.

## Introduction

Gene therapy has proven to be particularly successful in the eye [1, 2]. Although immunologic differences between the ocular and systemic compartments play a role [3], our laboratory has also shown that there are biochemical influences exerted by the vitreous of the eye that can modulate the expression of adenoviral vector delivered genes *in vitro* [4–6]. This paper will explore if the same and related biochemical pathways influenced by components of the vitreous similarly influence wild type adenovirus replication and gene expression and whether inhibitors of these pathways can modulate adenoviral gene expression in human tissue.

Adenoviruses are a family of medium-sized viruses that cause a wide variety of illnesses such as febrile respiratory disease, conjunctivitis, hemorrhagic cystitis, and hepatitis. These can trigger nervous system infections such as meningitis [7, 8]. Adenoviruses frequently cause severe disease in congenitally immunocompromised patients, especially in pediatric patients [8], as well as patients undergoing immunosuppressive treatment for organ and tissue transplants or cancers. Infections in these patients have an overall case fatality rate of 48%, and the virus is extremely resistant to a variety of physical and chemical agents [8, 9]. There is currently no effective cure for adenoviral infections.

Adenoviruses are often used as vectors for gene therapy after deleting their essential viral genes to render them replication-defective and replacing those genes with a cassette that expresses a foreign therapeutic gene [10]. It was previously discovered that *in vitro* transduction of cells by serotype 5 adenoviral vectors in the presence of vitreous, the gelatinous substance in the eye, significantly increases transgene expression [4]. This increase is partially due to a hyaluronan (HA)/CD44-mediated pathway. Sequential proteolysis of the hyaluronan receptor CD44 leads to this HA-mediated enhancement of gene expression; when HA binds CD44, it triggers matrix metalloprotease cleavage of CD44's extracellular domain. The remaining CD44 peptide then becomes the substrate of the γ-secretase complex, which cleaves CD44 again and liberates its intracellular domain. The intracellular domain translocates to the nucleus, where it can regulate gene expression. The inhibition of matrix metalloprotease and γ-secretase activities suppress the enhancement of adenoviral-mediated gene expression [5].

In this study, we show that these results are transferrable to wild-type adenoviruses serotype 5 (Ad5WT) that are responsible for common human infections, including severe infections in immunocompromised patients. Also, we demonstrate that inhibition of RhoA kinase [11] and protein kinase C [12], both of which have been linked to CD44-related biochemical pathways, can similarly inhibit both adenovector transgene expression (TGE) and Ad5WT replication. These findings could prove beneficial for the development of treatments for adenoviral infections and modulation of gene therapy protocols.

## Materials and methods

### Cell culture

Cell lines were cultured as follows: Both Y79-Rb (ATCC, HTB-18; Manassas, VA) and Hela cells (ATCC, CCL-2; Manassas, VA) were grown in DMEM (Mediatech, Manassas, VA) supplemented with 5% heat-inactivated bovine serum (Gemini Bio-Products, Sacramento, CA) and 1% Penicillin/Streptomycin. Cultures were maintained at 37°C with 5% CO_2_.

### Antibodies

The CD44 blocking antibody BRIC235 is a mouse monoclonal IgG2b antibody that was purchased from the International Blood Group Reference Laboratory (cat. 9407P) in the United Kingdom. The anti hexon antibody is a rabbit polyclonal to Adenovirus Type 5 hexon and was purchased from Abcam (cat. Ab24240). The anti-E1A antibody is a mouse monoclonal antibody to Adenovirus Types 2/5 E1A (clone M73) and was purchased from EMD Millipore Corporation (cat. 6B7386).

### Inhibitors

The γ-secretase inhibitor DAPT was purchased from Sigma-Aldrich (cat. D5942). The metalloproteinase inhibitors TAPI-0 (cat. 579050) and TAPI-1 (cat. 579051), the RhoA kinase (ROK) inhibitor Y-27632 (cat. 688000) and the protein kinase C (PKC) inhibitor Gö6983 (cat. 365251) were purchased from EMD Millipore Corporation.

### Adenoviral vectors

The first-generation adenoviral vectors delivering the luciferase gene (Luc) under the control of the cytomegalovirus promoter CMV (Ad5/CMV-Luc) or the green fluorescent protein (GFP) gene under the control of the CMV promoter (Ad5/CMV-GFP) were prepared and expanded by the Vector Development Laboratory at Baylor College of Medicine and stored in 25 μL aliquots at -80°C until needed.

### Wild type adenovirus

The wild type adenovirus type 5 (Ad5WT) was provided by Dr. Ann Leen, from the Center for Cell and Gene Therapy at Baylor College of Medicine and stored in aliquots at -80°C until needed.

### Adenovirus vector transduction or wild type adenovirus infection *in vitro*

Cells were counted using a hemocytometer and plated in a cell culture plate as indicated in the text using DMEM supplemented with 5% FBS and 1% antibiotics (complete growth media). Either a viral vector or wild type virus was diluted in complete growth medium to the appropriate concentration. Vitreous, inhibitors, antibodies, or diluent were mixed with the virus or vector to achieve the indicated final concentration. The mixture was overlaid on the cells. Cultures were kept at 37⁰C with 5% CO2 for either 24 or 48 hrs.

### Luciferase activity assay

To assay luciferase activity, cells dispensed in a 96-well plate (2 x 10^4^ cells/well) were washed once with PBS 1X. Once PBS 1X was removed, cells were lysed in 50 μL of Promega Lysis Buffer (Promega Corp). The plate was kept at -80°C for at least 15 minutes and then thawed slowly to room temperature. Next, 5 μL of cell lysate were added to 50 μL of luciferase substrate (Promega Corp.) and mixed gently by flicking the tube. The tube was placed in a luminometer, and light emitted was measured as counts per minute (CPM). CPMs were normalized to CPM/ μg lysate using protein concentrations determined using Bradford Assay Reagent (Bio-Rad).

### Adenovirus quantitative PCR (Ad-qPCR)

Cells were plated in 24-well plates (2 x 10^5^ cells/well) and transduced with the indicated multiplicity of infection (MOI) of wild type adenovirus or Ad5/CMV-Luc vector. Adenovirus was quantified by quantitative PCR [13] using the primer pairs and probes directed against a conserved sequence among different Ad serotypes within the hexon gene shown in **Table 1**.

**Table 1:** Q-PCR primer and probe sequences.

Name	Sequence (5' → 3')
Adenogene FWD	GCCACGGTGGGGTTTCTAAACTT
Adenogene REV	GCCCCAGTGGTCTTACATGCACATC
Adenogene Probe	56-FAM/TGCACCAGACCCGGGCTCAGGTACTCCGA/36-TAMSp
CRP FWD	CTTGACCAGCCTCTCTCATGC
CRP REV	TGCAGTCTTAGACCCCACCC
CRP Probe	56-FAM/TTTGGCCAGACAGGTAAGGGCCACC/36-TAMPs

The PCR reactions were set up and performed using Taqman 2X Universal Master Mix (Applied Biosystems, Carlsbad, CA) according to the manufacturer's directions, using primer concentration of 0.75 mM and a probe concentration of 0.5 mM. Q-PCR was conducted using the ABI Prism 7000 Sequence Detection System programmed as follows: 35°C for 2 minutes, 95°C for 10 minutes, and repeat 40 times: 95°C for 15 seconds, 55°C for 10 seconds. The copy numbers of adenovirus genomes were standardized to the number of cells quantified in the assay.

### Statistical analysis

Quantitative data were analyzed using the Graph Pad Prism 5 software. For comparison of only two averages, the Student's t-test was performed. For the comparison of more than two averages, a one-way ANOVA test was conducted followed by the Newman- Kleus Student t-test or the Dunnet's test to detect significant differences between all groups or between a control group and an experimental group. Statistical significance was considered when *p* ≤ 0.05. Symbols denoting levels of statistical significance are as follows: *, *p* = <0.05; **, *p*<0.01; and ***, *p*<0.001.

## Results

### Vitreous enhances Ad5 wild type (Ad5WT) replication and hexon expression

Previous reports have demonstrated that versican through both hyaluronan-CD44 dependent and independent mechanisms and their associated intracellular pathways can modulate adenoviral vector-mediated transgene expression [4–6]. Thus, it is reasonable to hypothesize that wild type adenoviral replication could also involve the same pathways.

**Fig 1:**
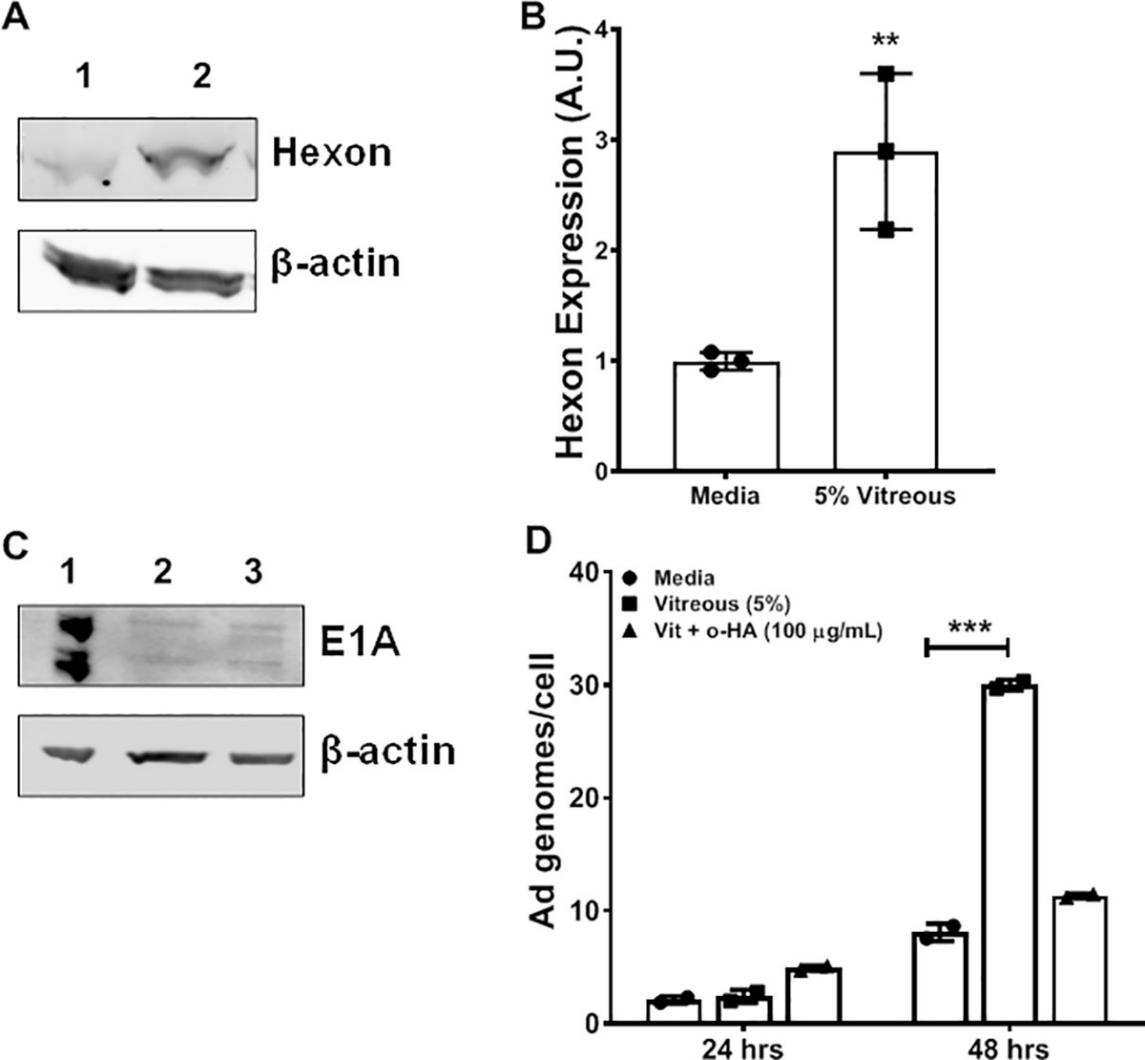
Vitreous enhances Ad5 wild type replication and hexon expression. **(A-C)** Y79 cells were infected in a 24 well plate (2x10^5^ cells/well) with Ad5WT (MOI: 250 genomes/cell) in the absence (A, Lane 1; B, lane 2) or presence (A, Lane 2; B, Lane3) of 5% vitreous. Total protein was isolated at 24 hours post-infection and analyzed for hexon (A) or E1A (B) expression by western blotting. Hexon levels were significantly increased after vitreous exposure (A-B) while E1A expression was unchanged (C). Lysate from HEK293 cells, which contain and express the Ad E1A gene, was used as a positive control (B, Lane 1). **(D)** Y79 cells were infected in a 24 well plate (2x10^5^ cells/well) with Ad5WT (MOI: 250 genomes/cell) in the presence or absence of 5% vitreous with or without small chain hyaluronic acid (o-HA) for 24 or 48 hours. Cells were harvested by centrifugation at 500 g for 10 minutes and trypsinized, followed by a wash with PBS 1X. Cell pellets were then stored at -80°C until all samples were collected. Total DNA was extracted using the DNEasy Kit (QIAGEN, Inc) as per the manufacturer's protocol. Ad genomes and cellular genomes were quantified using 125 ng of each sample in an Ad Q-PCR assay as described in Materials and methods. Ad genome numbers are standardized to cellular genome copies in each sample. Values represent the average ± standard deviation. (n = 4 biological replicates, p<0.0001).

To test this hypothesis, we first examined the effect of hyaluronan-containing vitreous on Ad5WT gene expression. If the CD44-HA pathway is involved, the incubation of the cells in the presence of vitreous should enhance the Ad5WT gene expression. We monitored the expression of the adenoviral coat protein hexon or replication regulatory protein E1A after incubation with vitreous by western blot (**Fig 1A–1C**). After 24 hours, the level of hexon protein expression is significantly increased but not the protein expression of E1A.

Next, we examined whether Ad5WT replication was affected by the presence of vitreous. Y79 retinoblastoma cells were infected *in vitro* with Ad5WT in the presence of vitreous. To measure adenoviral replication, an adenovirus-specific quantitative PCR was performed using DNA isolated from the cultured cells. After 48 hours, the addition of 5% vitreous to the cell cultures significantly increased the number of Ad genomes per cell by a 3.5-fold difference when compared to Ad genomes per cell in the absence of vitreous (**Fig 1D**). Incubation of the cells with short-chain HA oligosaccharides (o-HA) that antagonize HA–CD44 signaling blocked the increase in Ad genomes induced by the presence of vitreous [5]. These results show that, as hypothesized, the addition of vitreous can increase Ad5WT replication *in vitro*. However, we could not demonstrate an increase in active Ad5WT using a plaque-forming assay.

**Fig 2:**
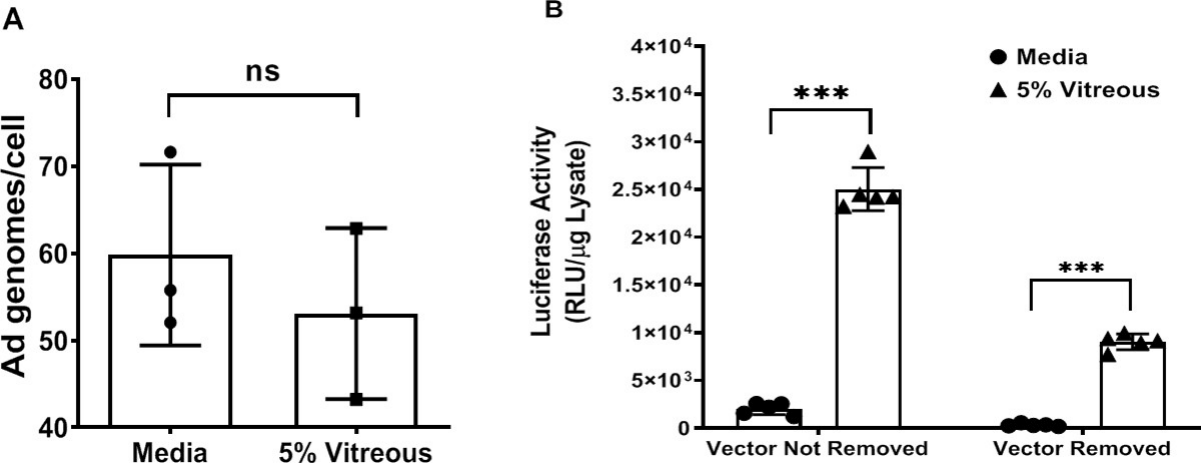
Vitreous enhances Ad TGE after virus binding/internalization. **(A)** Y79 cells were transduced in a 24 well plate (2x10^5^ cell/well) with Ad5/CMV-*Luc* (MOI: 250 pfu/cell) in the presence or absence of 5% vitreous for 1 hr at 37°C. Cells were then trypsinized and washed once with PBS 1X followed by a 10 minutes centrifugation (500 g) at 4°C. DNA was extracted from cell pellets using the DNEasy kit from QIAGEN as per the manufacturer's procedure. Adenoviral vector genomes were quantified using the adenovirus quantitative PCR technique as stated in Materials and Methods. (n = 3 biological replicates, p = 0.46, n.s. = not significant by student t-test) **(B)** Y79 cells were transduced with Ad5/CMV-*Luc* (MOI: 250 pfu/cell) in the presence or absence of 5% vitreous in a 96 well plate (2x10^4^ cell/well). In the "non-removed" group, the vector was present during the whole procedure. In the "removed" groups, cells were transduced with the same vector for 1 hr at 37°C before being washed once with complete media and then incubated with media alone or with 5% vitreous for the remainder of the assay. Cells were then lysed, and luciferase activity was determined as stated in Materials and Methods. Values represent the average ± standard deviation. (n = 5 biological replicates, p<0.0001).

A potential explanation for the vitreous mediated enhancement of Ad5WT replication and gene expression could be due to vitreous increasing the number of viral particles being internalized by the cells. This hypothesis was tested by quantifying the number of genomes after transduction with an Ad5 vector delivering the luciferase gene. This vector provides the same capsid proteins as the Ad5WT while allowing a more sensitive method to further study changes in gene expression. We trypsinized cells after 1-hour incubation with our vector in the presence or absence of 5% vitreous humor to remove any bound but not internalized viral particles. Our results showed that the simultaneous incubation of our Ad5 vector with vitreous did not increase the number of vector genomes detected within the cells (**Fig 2A**). To further test this hypothesis, we compared the effect of adding Ad5 in the presence of vitreous or adding vitreous after removing the virus. As expected, co-incubation of vitreous with the Ad5 vector increased the level of transgene expression; however, the addition of vitreous after vector removal of the Ad5 vector also caused an increase in transgene expression (**Fig 2B**). Together these results demonstrate that the vitreous enhancement of viral replication and or gene expression is unlikely due to an increase in the virus or vector transduction/infection.

### Inhibition of matrix metalloproteases or the γ-secretase complex decreases Ad5WT replication *in vitro*

**Fig 3:**
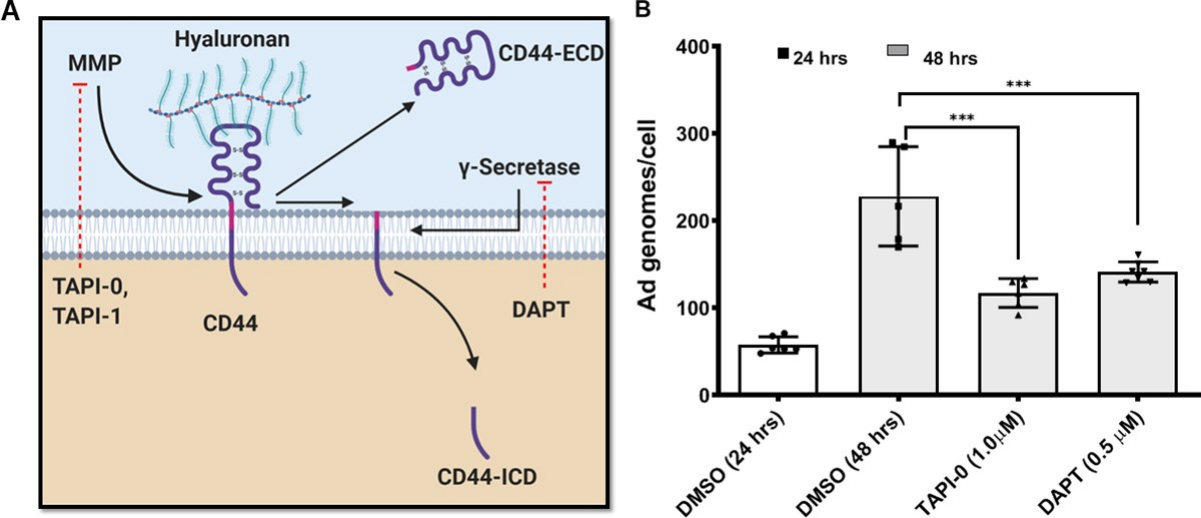
Inhibition of metalloproteases or the γ-secretase complex inhibits Ad5 wild type replication *in vitro*. **(A)** Hyaluronan binding to its receptor CD44 is followed by sequential proteolysis first by MMP (inhibited by TAPI-0 or TAPI-1) to release the extracellular domain followed by proteolysis by the γ-secretase complex (inhibited by DAPT) to release the intracellular domain. **(B)** Y79 cells were infected in a 24 well plate (2x10^*5*^ cells/well) with Ad5WT (MOI: 250 genomes/cell) in the presence of DMSO (1:10^*3*^ dilution) for 24 hours, or with DMSO (1:10^*3*^ dilution), TAPI-0 (1 μM), or DAPT (0.5 μM) for 48 hours. Cells were harvested by spinning at 500 g for 10 minutes and trypsinized, followed by a wash with PBS 1X. Cell pellets were then stored at -80°C until all samples were collected. Total DNA was extracted using the DNEasy Kit (QIAGEN, Inc.) as per the manufacturer's protocol. Ad genomes and cellular genomes were quantified using 125 ng of each sample in an Ad Q-PCR assay as described in Materials and methods. Ad genome numbers are standardized to cellular genome copies in each sample. Values represent the average ± standard deviation. (n = 6, p<0.0001).

The vitreous enhancement of Ad5WT replication and its inhibition by o-HA *in vitro* strongly suggests that the same CD44-dependent mechanism involving sequential proteolysis of CD44 in response to hyaluronan that modulates Ad TGE (**Fig 3A**) could also affect wild type virus replication *in vitro*. Cells were infected in the presence or absence of the MMP inhibitor TAPI-0 or the γ-secretase inhibitor DAPT for 48 hours at 37°C to test this hypothesis. Results show that at 48 hours, there is viral replication in the presence of DMSO solvent (control) (**Fig 3B**). However, cells that were infected in the presence of either TAPI-0 or DAPT showed a significant reduction in the amount of replication when compared to the DMSO 48 hours control group. From these results, it is concluded that the inhibition of the MMP activity or the γ-secretase complex activity can significantly decrease Ad5WT replication *in vitro*.

### Inhibition of matrix metalloproteases or the γ-secretase complex inhibits Ad TGE *in situ*

**Fig 4:**
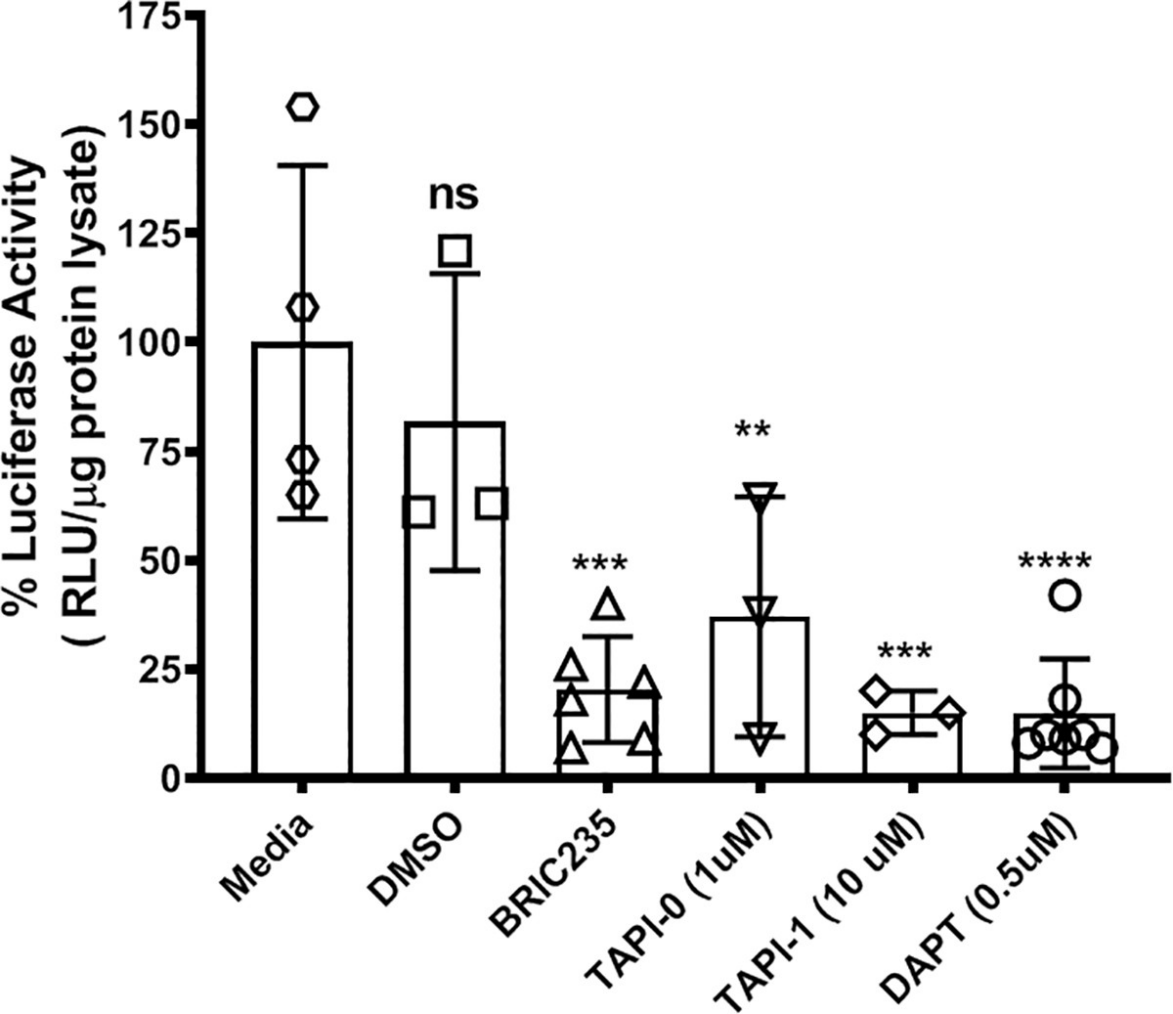
Inhibition of CD44, matrix metalloproteases, or the γ-secretase complex *in situ* inhibits Ad TGE. Human conjunctiva explants were obtained from the Lions Eye Bank donated eyes. Punch biopsies of the conjunctiva were placed in the wells of 96 well plates and transduced with Ad5-CMV-Luc (1.25x10^7^ pfu/well) in the presence or absence of the CD44 blocking antibody BRIC235 (4 μg/well), the MMPs inhibitors TAPI-0 (1 μM) or TAPI-1 (10 μM), or the γ-secretase inhibitor DAPT (500 nM) for 16 hrs. Samples were lysed, and luciferase activity determined as described in Materials and Methods. Values were standardized to luciferase activity of media control samples. Values represent the average ± standard deviation. (n = 2 eye pairs, *** = p<0.001).

The experiments described in this manuscript have been performed *in vitro*. The Ad5WT used in these experiments can only replicate in human tissues and there is no accurate animal model of human adenovirus conjunctivitis disease. We, therefore, examined whether Ad TGE was affected by the MMP and γ-secretase inhibitors in human conjunctiva explants, a tissue commonly targeted by wild type adenovirus that results in conjunctivitis (**Fig 4**). Human conjunctiva explants from cadaver eyes were obtained from the Lions Eye Bank. Punch biopsies of equal size were obtained using a trocar and placed in 96 well plates. Each biopsy was transduced with Ad5-CMV-*Luc* (1.25 x 10^7^ pfu/well) in the presence or absence of the CD44 blocking antibody BRIC235 (4 μg/well), the MMP inhibitors TAPI-0 (1 μM)) or TAPI-1 (10 μM), or the γ-secretase inhibitor DAPT (500 nM) for 16 hours. Samples were lysed, and luciferase activity determined as described in Materials and methods. These inhibitors decreased TGE *ex vivo* in a human tissue where adenovirus can cause disease.

### Activation of protein kinase C (PKC) regulates the vitreous enhancement of Ad TGE

**Fig 5:**
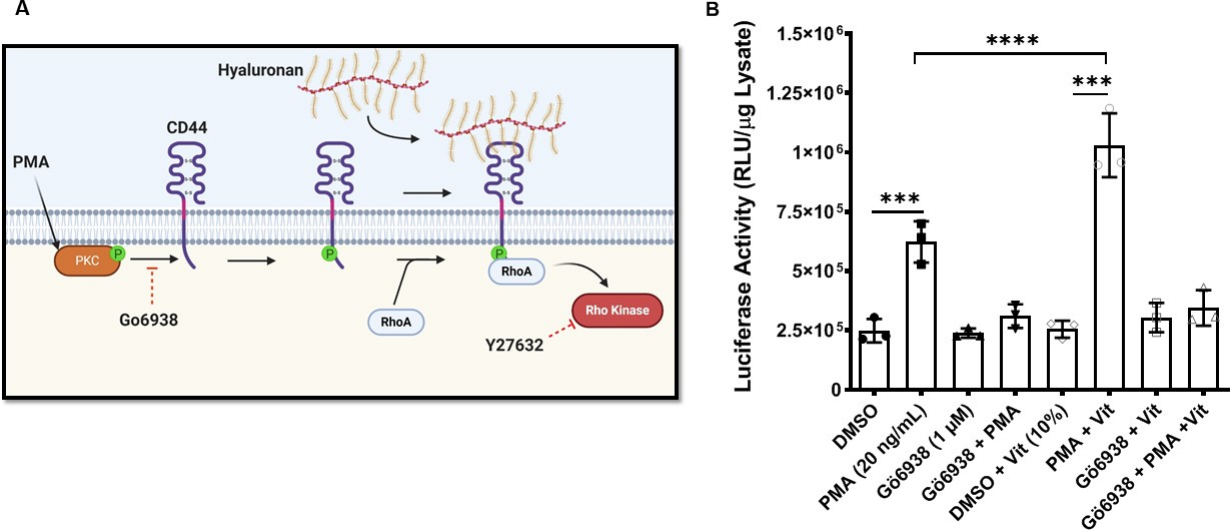
Activation of PKC is needed for the vitreous enhancement of Ad TGE. **(A)** CD44 is phosphorylated by activated PKC within its intracellular domain. This increases the affinity of CD44 for its ligand hyaluronan (HA). HA-engaged CD44 can then recruit RhoA, which in turn activates Rho-kinase. PMA activates PKC, while Go6938 inhibits it. The small molecule Y27632 inhibits the activity of Rho-kinase. **(B)** HeLa cells were seeded in a 96 well plate at 2x10^4^ cells per well. The next day, cells were incubated with either 100μL of DMSO (1:10^3^), 100μL of PMA (20 ng/mL) alone, 100 μL of the PKC inhibitor Gö6983 (1μM) alone, or 100 μL of both compounds for 1 hr at 37°C. The cells were washed and then transduced with Ad5/CMV-*Luc* (MOI: 50 pfu/cell) in the presence or absence of 10% vitreous for 18 hrs. Cells were then lysed, and luciferase activity determined as described in Materials and Methods. Values represent the average ± standard deviation. (n = 8, p<0.0001 by Dunnett's test).

We have previously shown that phosphorylation of the intracellular domain of CD44 by calmodulin-dependent protein kinase II (S325) and, to a lesser degree, protein kinase C (PKC, S291) is essential in the enhancement of Ad TGE by vitreous [5]. The model of Ad TGE modulation predicts that the inhibition of PKC might also potentially decrease viral replication (**Fig 5A**). The phosphorylation of the cytoplasmic domain of CD44 by PKC increases its ability to bind hyaluronan. To test our model, we pharmacologically activated or inhibited PKC activity in the presence of vitreous. Cells were incubated with or without 5% vitreous in the presence or absence of DMSO, the PKC activator phorbol 12-myristate 13-acetate (PMA), or the PKC inhibitor Gö6938, or their combination. These cells were transduced with the Ad5 vector delivering the luciferase transgene. Our results demonstrate that PMA activation of PKC increases transgene expression, which can be inhibited by the co-incubation with Gö6938 (**Fig 5B**). Furthermore, the presence of PMA allows for the vitreous enhancement of transgene expression, which is abrogated in the presence of the PKC inhibitor. Together, these results indicate that activation of PKC is necessary for the hyaluronan-CD44 mediated enhancement of Ad transgene expression.

### Inhibition of RhoA kinase (ROK) decreases vitreous enhancement of Ad TGE

**Fig 6:**
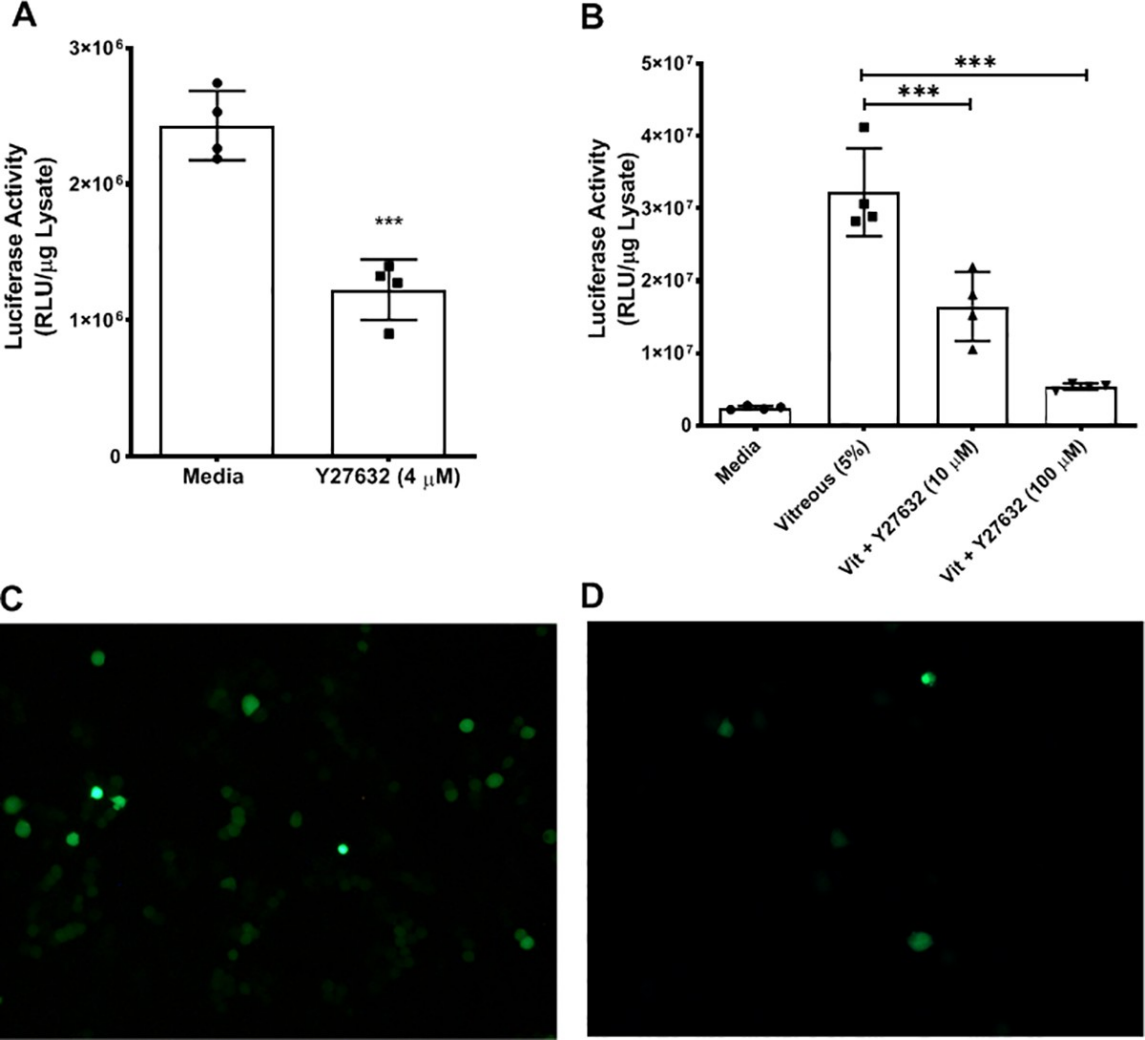
Inhibition of RhoA kinase (ROK) decreases vitreous enhancement of Ad TGE and vitreous enhancement of TGE. **(A)** Effect of the ROK inhibitor Y-27632 on Ad TGE. Y79 cells were transduced with Ad5-CMV- Luc (MOI: 250 pfu/cell) in the presence or absence of Y-27632 (4 μM) for 16 hours. Cells were lysed, and luciferase activity determined as described in Materials and Methods. (n = 4, p<0.0001) **(B)** Effect of the inhibitor Y-27632 on the vitreous enhancement of Ad TGE. Y79 cells were transduced with Ad5-CMV-Luc (MOI: 250 pfu/cell) in the presence or absence of 5% vitreous with or without different doses of Y-27632 for 16 hours. Cells were lysed, and luciferase activity determined. Values represent the average ± standard deviation. (n = 5, ** = p<0.01, *** = p<0.001) **(C, D)** Effect of the inhibitor Y-27632 on the Ad TGE using eGFP. Y79 cells were transduced with Ad5- CMV-eGFP (MOI: 103 pfu/cell) in the absence **(C)** or presence **(D)** of the Y-27632 for 16 hours. Images are representative fluorescent microscopic fields.

The interaction of CD44 with its ligand HA is known to activate several intracellular pathways. RhoA kinase (ROK) is a signaling partner that interacts with the cytoplasmic domain of the CD44 receptor upon engagement with its ligand HA [11]. Our model in Fig 5A predicts that inhibition of ROK can inhibit vitreous mediated enhancement of transgene expression. To determine if ROK activity is involved with Ad TGE, the effect of ROK inhibitor Y-27632 on Ad TGE was examined *in vitro*. Y79 cells were transduced with a first-generation adenoviral vector delivering the luciferase gene in the presence or absence of 5% vitreous with or without the ROK inhibitor Y-27632 (**Fig 6A** and **6B**). The inhibition of ROK activity in the absence of vitreous results in a significant decrease in Ad TGE. Furthermore, the inhibition of ROK in the presence of vitreous results in a significant decrease in the enhancement of Ad TGE in a dose-dependent manner.

A similar experiment was conducted using a first-generation adenoviral vector to deliver the fluorescent eGFP transgene to demonstrate that this effect is not transgene specific. Images show similar results to those obtained when using a luciferase transgene; inhibition of ROK results in a decrease in transgene expression (**Fig 6C** and **6D**). The results of these experiments suggest that the activity of ROK is required for the Ad TGE in the presence or absence of vitreous. The observation that the ROK inhibitor was capable of decreasing Ad TGE in the absence of vitreous suggests that this kinase also modulates the baseline expression of the adenoviral transgene. Nonetheless, the presented data would suggest that this could be a potential molecular target for the modulation of Ad TGE.

### Inhibition of protein kinase C or RhoA kinase (ROK) Inhibits Ad5WT replication *in vitro*

**Fig 7:**
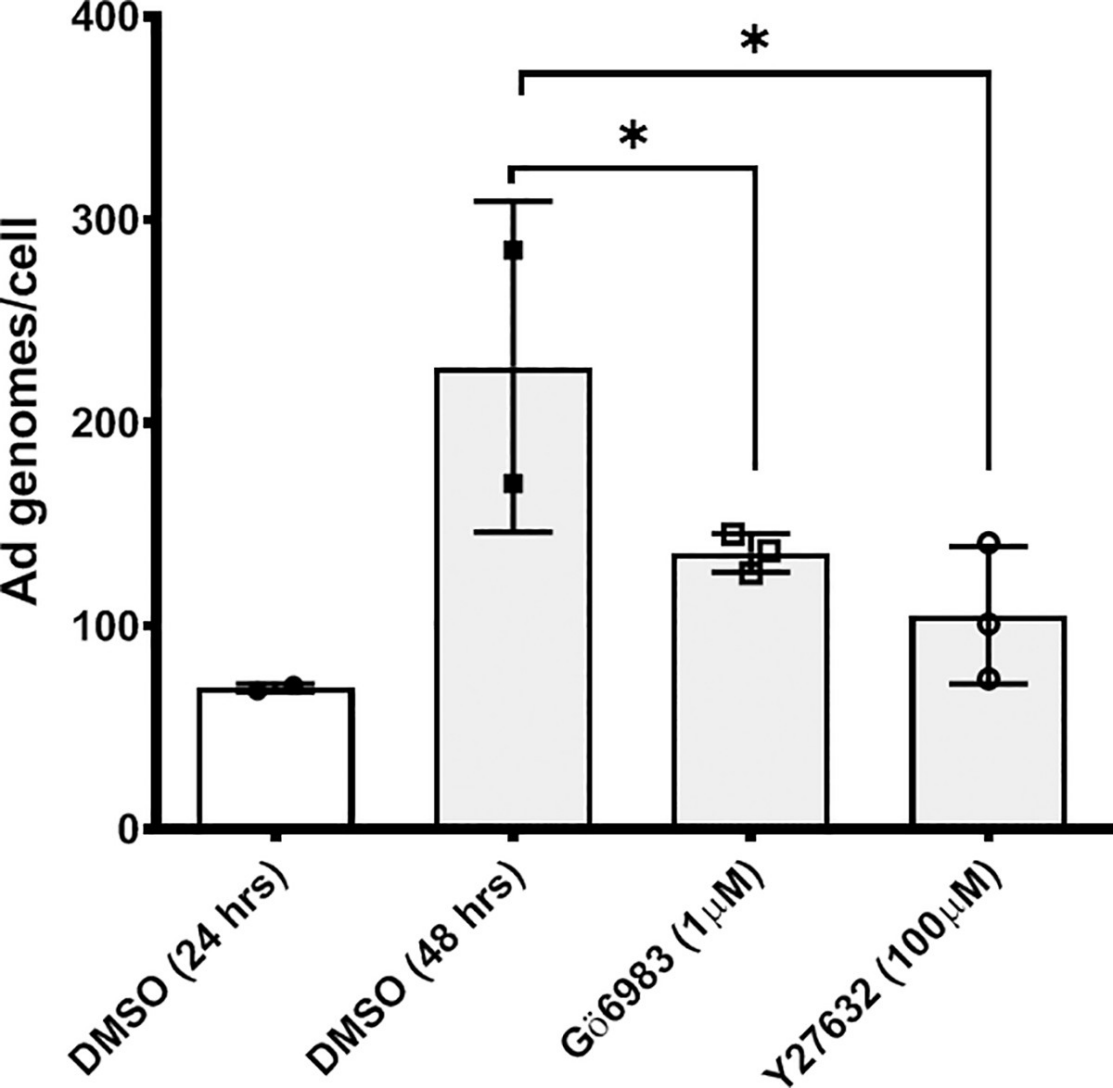
Inhibition of PKC or Rho protein kinase decreases Ad5 wild type replication *in vitro*. Y79 cells were infected in a 24 well plate (2x10^5^ cells/well) with Ad5WT (MOI: 250 genomes/cell) in the presence of DMSO (1:10^3^ dilution) for 24 hours, or with DMSO (1:10^3^ dilution), or with Gö6983 (1 μM), or with Y27632 (100 μM) for 48 hours. Cells were harvested by centrifugation at 500 g for 10 minutes and trypsinized, followed by a wash with PBS 1X. Cell pellets were then stored at -80°C until all samples were collected. Total DNA was extracted using the DNEasy Kit (QIAGEN, Inc) as per the manufacturer's protocol. Ad genomes and cellular genomes were quantified using 125 ng of each sample in an Ad Q-PCR assay as described in Materials and methods. Ad genome numbers are standardized to cellular genome copies in each sample. Values represent the average ± standard deviation. (n = 6 biological replicates, p<0.0001).

Given our observation that both PKC and ROK can be targeted to modulate adenoviral vector transgene expression, we hypothesize that inhibition of either of these enzymes can decrease the replication of Ad5WT. Cells were infected with Ad5WT in the presence or absence of the PKC inhibitor Gö6983 to test this hypothesis. A single dose of either inhibitor or its solvent DMSO alone was added on day zero, and cells were cultured for an additional 24 or 48 hours. The addition of the PKC or ROK inhibitor was sufficient to significantly decrease Ad5WT replication after a 48 hour incubation period (**Fig 7**). These results further support the similar hypothetical model through which Ad TGE and Ad5WT replication can be modulated through the modulation of PKC or ROK activity.

## Discussion

Adenoviral infections occur both in healthy human hosts as well as in immunocompromised patients [7]. Most adenoviral infections in immunocompetent hosts are self-limiting and only require supportive treatment. Adenovirus infections can be fatal in neonates and immunocompromised individuals as well as rarely in otherwise normal adults and children. In these affected patients antiviral agents have been used. Cidofovir is the most frequently used pharmaceutical to treat life-threatening adenoviral infections but severe nephrotoxicity is dose-limiting. Published data are limited to anecdotal case reports and small non-randomized studies but treatment has been associated with clinical improvement and a suggestion of increased survival in recipients of human stem cell and lung transplants infected by adenovirus [14, 15]. Brincidofovir is an oral lipid ester of cidofovir and is an investigational drug reported to have less nephrotoxicity [16]. Pooled Intravenous gamma globulin that contain high titers of antibody to adenovirus is used in patients with hypogammaglobulinemia and may have some effect in patients with adenoviral infection [17]. Immunotherapy with donor lymphocytes stimulated with adenovirus *in vitro* were used with some success when injected into human stem cell recipients infected with adenovirus [18, 19]. We propose potential targets for these infections. The observation described in this study that PKC, ROK, MMP, and γ-secretase inhibitors that have previously been described to modulate transgenic Ad5 gene expression [5] also inhibited wild-type Ad5 gene expression.

Matrix metalloprotease inhibitors like TAPI-0 and TAPI-1 have been studied in clinical trials as metastatic cancer treatments [20, 21]. Although with limited success, these studies provide data on the safety and tolerability of these drugs. Similarly, the field of Alzheimer's Disease has completed multiple clinical studies evaluating the effects of γ-secretase in disease progression. Although toxicity problems have been reported with these inhibitors due to its interference with Notch signaling, new compounds that inhibit the γ-secretase complex while sparing Notch signaling are under development [22]. Studies focusing on the use of PKC inhibitors for diabetic retinopathy [23–25] have demonstrated the tolerability of these molecules and potential efficacy in the disease. Finally, the inhibition of ROK for ocular disease using small compounds have also been explored in subjects with diabetic macular edema [26]. These studies provide evidence of the potential clinical value of targeting these pathways and, based on our studies, could indicate a potential new repurposing of these compounds. Future studies will be needed to determine the efficacy of repurposing these compounds for adenovirus infections.

The sequential proteolysis and liberation of CD44 intracellular domain has been described [27]. Once liberated, CD44-ICD can regulate gene expression through three hypothetical mechanisms: transactivation, tethering, or synergism [28]. CD44-ICD transactivation is believed to be mediated through a direct interaction of CD44-ICD with its response element (CIRE: CCTGCG), which can be found 38 times throughout the Ad5 genome. Although not tested in our work, we can predict that CD44-ICD could be in part, mediating increase Ad5 gene expression through transactivation. This CIRE sequence is absent from the CMV promoter used in our studies, which suggest that CD44-ICD could be working through a tethering mechanism requiring a specific transcription factor. Future studies will test our hypothesis concerning CD44-ICD mechanism of action.

Previous research has demonstrated that gene therapy can potentially benefit patients with ocular diseases [1, 2]. We have explored mechanisms both immunologic [3] and biochemical [4–6] that can explain these results. The mechanism of anterior chamber acquired immune deviance (ACAID) can explain the long duration of transgene expression of cells within the ocular environment [29, 30]. Our laboratory has also found that vitreous, the gelatinous material within the globe of the eye, can enhance transgene expression delivered by adenoviral vectors. There are both hyaluronan dependent and independent mechanisms and mechanisms that involve versican [5, 6]. Both versican and hyaluronan are expressed in relatively high concentrations in vitreous [31, 32]. The hyaluronan dependent mechanism appears to involve the hyaluronan receptor CD44. When hyaluronan binds to CD44, metalloproteinase cleaves the extracellular domain creating the substrate that allows γ-secretase to release an intracytoplasmic peptide that translocates to the nucleus [33, 34]. This second proteolytic step appears to require PKC phosphorylation of the intracytoplasmic domain adjacent to the proteolytic domain [35]. When inhibitors of these proteases and the kinase are introduced *in vitro*, the enhancement of adenoviral-mediated transgene expression is inhibited [5]. Downstream biochemical events that are influenced by CD44 include RhoA kinase-related pathways [36]. Inhibitors of RhoA kinase also inhibit vitreous-enhanced adenoviral-mediated transgene expression. This process appears to be under the influence of Src kinase since Src inhibitors greatly enhance adenoviral-mediated transgene expression [6].

To identify potential targets for therapeutic intervention in wild-type adenoviral infection, we explored whether each of these modulators of adenoviral vector transgene expression could affect wild-type F5 adenoviral replication. First, we found that incubation with vitreous enhanced adenoviral hexon expression and replication. Interestingly, we could not verify an increase in active viral infectious particles using a standard plaque-forming assay. Potential explanations for this could be a lack of sensitivity of the assay or failure of our *in vitro* system to produce more active infectious units even though increased hexon and viral DNA had been produced. Importantly, specific proteolytic and kinase inhibitors inhibited adenoviral replication, therefore, implicating these pharmacologic agents as potential therapeutic options to treat and prevent adenoviral infections, a leading cause of human morbidity and in severely immunocompromised individuals, mortality. Furthermore, these same inhibitors could inhibit Ad TGE in human conjunctiva a target tissue of wild-type adenoviral infection.

Since transcription control appeared to be involved in the transgene expression [5], transcription regulatory pathways were next explored. A previous study has implicated Janus kinase (JAK) activity being required for vitreous-mediated TGE enhancement [6]. When the JAK1/2 inhibitor ruxolitinib was added to Ad5-transduced cells incubated in vitreous or versican containing media (VCS), the vitreous or VCS-mediated enhancement of transgene expression was inhibited. The same effect was observed when STAT3/5 inhibitors C188-9 and 5,15-DPP were employed [6]. Although this has not yet been tested in cells transduced with Ad5WT, it is possible that JAK/STAT can be another target to treat adenoviral infections.

There currently is a need for pharmacologic preventive or therapeutic regimens for adenoviral infections. We have previously found biochemical pathways that modulate transcription of transgenes delivered by adenoviral vectors previously used in gene therapy. This manuscript demonstrates that these same biochemical targets might be useful to treat and prevent wild type adenoviral infections in humans.

## Supporting information

S1 Fig Y79 WT Ad5 hexon western. Full western blot in support of Fig 1A.
(TIF)

S2 Fig Y79 WT Ad5 E1a western. Full western blot in support of Fig 1C.
(TIF)
