# Supplementary figures and images for: Inhibitors of metalloprotease, γ-sectretase, protein kinase C and Rho kinase inhibit wild-type adenoviral replication
Source: PLoS One. 2020 Jul 22;15(7):e0236175. doi: 10.1371/journal.pone.0236175 (PMC7375579; doi:10.1371/journal.pone.0236175)

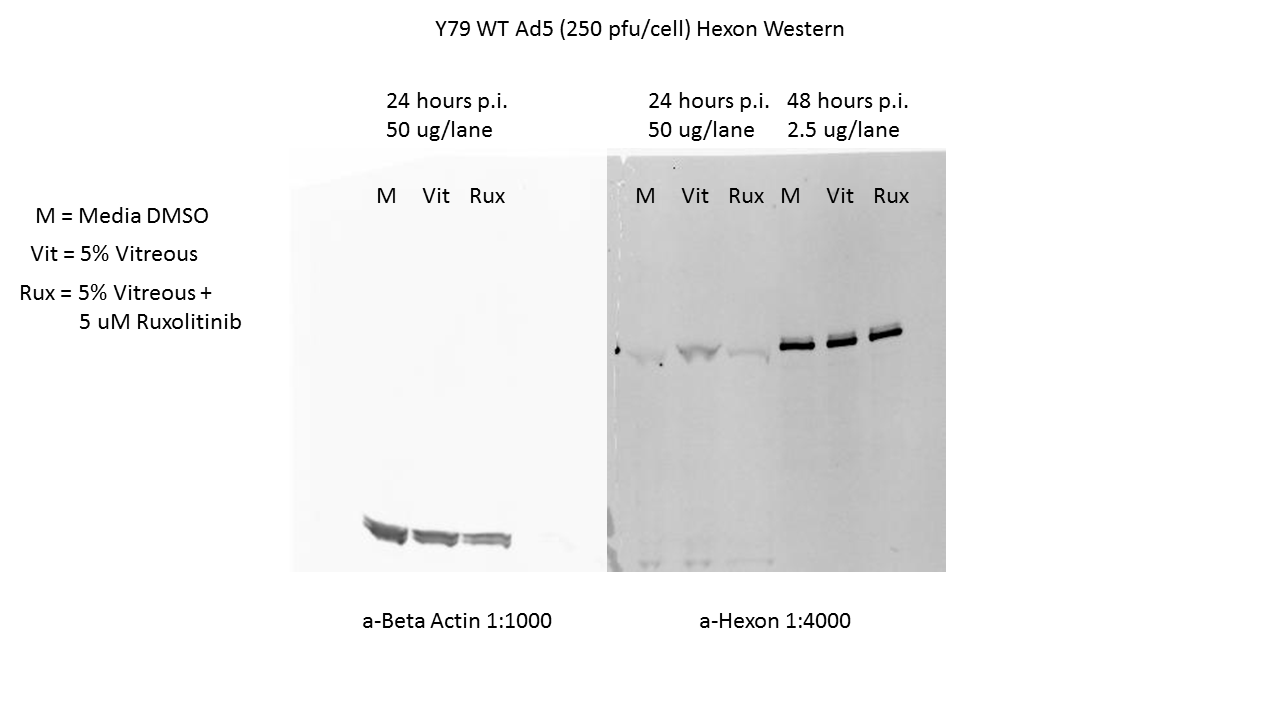

Supplement: S1 Fig — Full western blot in support of Fig 1A. (TIF) [file pone.0236175.s001.tif]

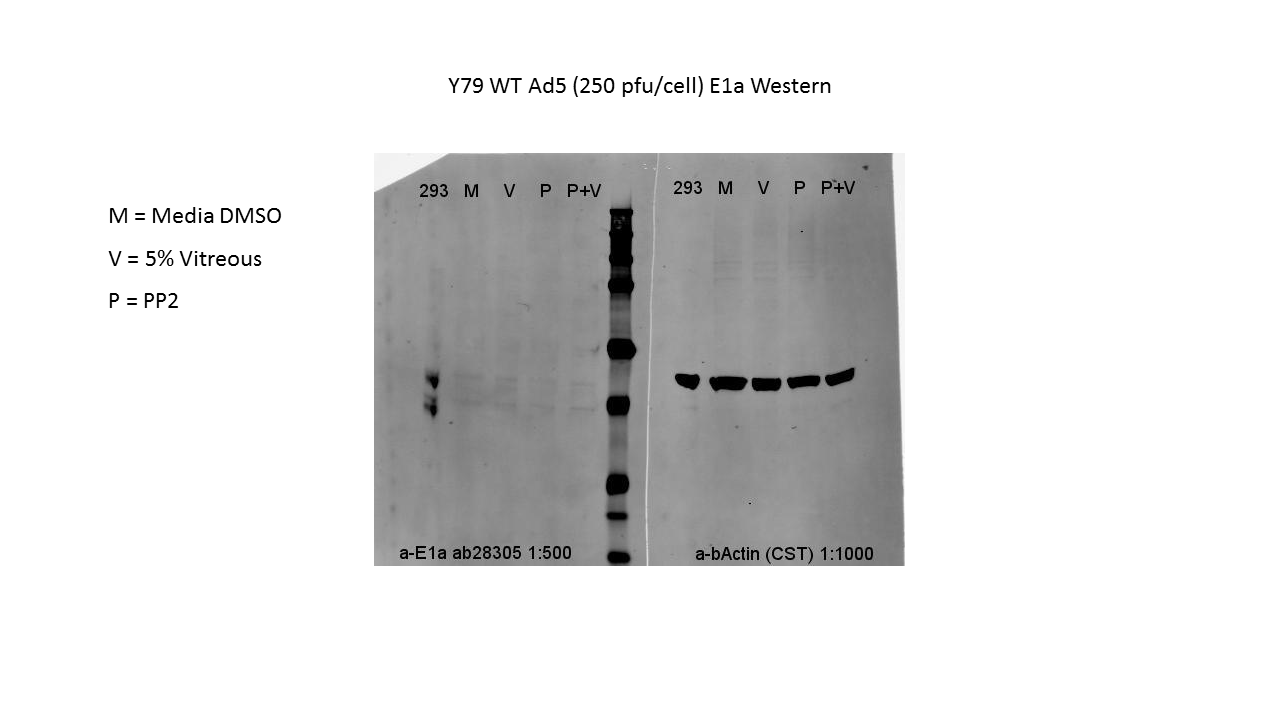

Supplement: S2 Fig — Full western blot in support of Fig 1C. (TIF) [file pone.0236175.s002.tif]
